# Cytotoxicity Effects of *Amoora rohituka* and *chittagonga* on Breast and Pancreatic Cancer Cells

**DOI:** 10.1155/2011/860605

**Published:** 2011-04-26

**Authors:** Leo L. Chan, Sherine George, Irfan Ahmad, Saujanya L. Gosangari, Atiya Abbasi, Brian T. Cunningham, Kenneth L. Watkin

**Affiliations:** ^1^Department of Electrical and Computer Engineering, University of Illinois at Urbana-Champaign, Urbana, IL 61801, USA; ^2^Department of Bioengineering, University of Illinois at Urbana-Champaign, Urbana, IL 61801, USA; ^3^Micro and Nanotechnology Laboratory, University of Illinois at Urbana-Champaign, Urbana, IL 61801, USA; ^4^Bioimaging Science and Technology Group, Beckman Institute for Advanced Science and Technology, University of Illinois at Urbana-Champaign, Urbana, IL 61801, USA; ^5^College of Applied Health Science, University of Illinois at Urbana-Champaign, Champaign, IL 61820, USA; ^6^Medical Imaging Research Laboratory, Department of Speech and Hearing Science, University of Jllinois at Urbana-Champaign, Urbana, IL 61801, USA; ^7^International Center for Chemical and Biological Sciences, University of Karachi, Karachi 75270, Pakistan

## Abstract

Chemotherapeutic agents for cancer are highly toxic to healthy tissues and hence alternative medicine avenues are widely researched. Majority of the recent studies on alternative medicine suggested that *Amoora rohituka* possesses considerable antitumor and antibacterial properties. In this work, *rohituka* and *chittagonga*, fractionated with petroleum ether, dichloromethane, and ethanol, were explored for their anticancer potential against two breast cancer (MCF-7 and HTB-126) and three pancreatic cancer (Panc-1, Mia-Paca2, and Capan1). The human foreskin fibroblast, Hs68, was also included. Cytotoxicity of each extract was analyzed using the MTT assay and label-free photonic crystal biosensor assay. A concentration series of each extract was performed on the six cell lines. For MCF-7 cancer cells, the *chittagonga* (Pet-Ether and CH_2_Cl_2_) and *rohituka* (Pet-Ether) extracts induced cytotoxicity; the *chittagonga* (EtoAC) and *rohituka* (MeOH) extracts did not induce cytotoxicity. For HTB126, Panc-1, Mia-Paca2, and Capan-1 cancer cells, only the *chittagonga* CH_2_Cl_2_ extract showed a significant cytotoxic effect. The extracts were not cytotoxic to normal fibroblast Hs68 cells, which may be correlated to the specificity of *Amoora* extracts in targeting cancerous cells. Based on these results, further examination of the potential anticancer properties *Amoora* species and the identification of the active ingredients of these extracts is warranted.

## 1. Introduction

Cancer is one of the leading causes of death in the United States, which is resulted by the uncontrollable division of abnormal cells. Specifically, breast cancer is one of the most common cancers worldwide with high mortality rate if diagnosed in the later stages. However, if discovered at an early stage and with proper treatments, these cancerous cells could be completely removed [1, 2]. Pancreatic cancer has a high mortality rate and often cannot be detected at an early stage due to the fact that symptoms do not appear until the disease has advanced significantly [3, 4]. Typical treatment regimes include targeting the tumor with ionizing radiation, surgical removal of tumor tissue, and chemotherapy. However, these current cancer treatment methods also cause severe systemic side effects. For this reason, recent research has focused on the search for alternative medicines extracted from plant-based sources. The use of alternative medicines, especially when used in conjunction with conventional cancer treatments, can serve to mitigate the side effects [5], enhance the uptake of conventional medicines, and, bolster the immune system to fight the cancer. Since these medicines are primarily extracts of naturally occurring flora, their bioavailability is less likely to induce severe immune responses. 

Several species of the *Amoora* plant extract in many parts of Bangladesh possess a multitude of medicinal properties against inflammation, cancer, and diseases of the liver [6–8]. One of the most common and the most studied *Amoora* species, the *Amoora rohituka*, is an evergreen tree that grows wildly in the region and is planted in many districts of Bangladesh [9]. It is traditionally used as herbal medicine for cancer, tumor, liver, and spleen diseases. The petroleum ether and methanol extracts of *Amoora rohituka* are reported to possess good laxative potential and can be developed to perform as safer gastrointestinal agents [10]. Furthermore, *Amoora rohituka* extracts are also known to possess antimicrobial activity [11] (with an IC_50_ at ~360 *μ*g/mL). The plant triterpenic acid, amooranin, extracted from the bark of *Amoora rohituka* trees, has been reported to possess significant anticancer potential [7, 12]. In the earlier studies, amooranin has been shown to induce apoptosis in breast carcinoma through caspase activity [13] and is known to be effective against breast cancer, colon cancer, cervical cancer, and leukemia cell panels [14]. Besides anticancer activity, amooranin is also known to induce the reversal of multidrug resistance in human leukemia and colon cancer cells lines [15]. An amooranin concentration of 10 *μ*M or higher has been shown to cause a cytotoxic response. One of the amooranin derivatives, amoorastatin, has shown significant inhibition activity against murine P388 lymphocytic leukemia cell lines [16]. Furthermore, chromone alkaloid rohitukine, a leading compound isolated from *Amoora rohituka,* has shown anticancer effect on non-Hodgkin's lymphoma as well as renal, prostate, colon, and gastric cancers [17]. In addition, the rocaglamide derivatives of the *Amoora cucullata* species have been reported to possess potent antitumor activity against KB, BC, and NCI-H187 cancer cell lines [18]. Currently, little is known about the bioactivity of *Amoora chittagonga* except that it is present in a number of south eastern Asian countries, such as Bangladesh and Thailand [19, 20]. The plant extracts explored in this research are from the *Mahogany* family. The *Meliaceae*, or the *Mahogany* family, is a flowering plant family of mostly trees and shrubs (and a few herbaceous plants) in the order Sapindales.

The majority of reports have investigated medicinal properties of *Amoora rohituka*, but it is important to explore the effects of other less-studied species of *Amoora* on various cancer cell lines. In this work, two species of the genus *Amoora*, *rohituka* and *chittagonga*, were studied for their anticancer potential on a panel of five human cancer cell lines and one human foreskin fibroblast cell line. This panel consisted of the following cell lines: MCF-7 and HTB-126 (breast cancer), Panc-1, Mia-Paca2, and Capan1 (pancreatic cancer), and Hs68 (fibroblast cell). 

The *Amoora chittagonga *was fractionated with pet-ether (petroleum ether), CH_2_Cl_2_ (dichloromethane), and EtoAC (ethanol), while *Amoora rohituka* was fractionated with pet-ether and MeOH (methanol). Utilizing a conventional MTT assay, the five extracts were tested on each cell line at four concentrations ranging from 100 *μ*g/mL to 0.1 *μ*g/mL and the IC_50_ values were determined. The results of the MTT assay were then confirmed using a label-free photonic crystal (PC) biosensor assay. The PC biosensor provides an image of the attachment of cells on the sensor surface before and after the incubation with plant extracts, which can determine the cytotoxicity effects [11, 21]. These biosensors are incorporated into the bottom of a 96- and 384-well standard microplate, which enables high-throughput screening of multiple extracts and cancer cells lines simultaneously and allows the rapid characterization of potential cytotoxic compounds. 

## 2. Materials and Methods

### 2.1. Plant Extracts

The extracts, *Amoora chittagonga* and *Amoora rohituka,* were provided by Dr. Chowdhury from the University of Dhaka at Bangladesh. Both *Amoora rohituka* and *chittagonga *stem bark was collected from Comilla, which is in the Chittagong Division, Comilla District of Bangladesh [22].

 The plants were identified at the Bangladesh National Herbarium, where voucher specimens have been deposited under the accession numbers DACB-28927 [22]. The air-dried and powdered stem bark (507.6 g) was successively extracted with light petroleum ether (40°–60°), dichloromethane, and methanol in a Soxhlet apparatus at an elevated temperature. All three extracts were filtered through fresh cotton beds. The filtrates were evaporated under reduced pressure at 40°C using a Büchi rotary evaporator to have gummy concentrates of petroleum ether (6.6 g), dichloromethane (4.3 g), and methanol (11.6 g) extracts.

Stock solutions of the dried extracts were prepared by dissolving them in ethanol to a concentrations of 25 mg/mL or higher. They were diluted directly in cell culture media and tested on the cancer cell lines at concentrations of 10, 25, 50, and 100 *μ*g/mL. Testing on normal cell line was performed at concentrations of 0.1, 1, 10, and 100 *μ*g/mL, in order to test for the absence of cytotoxicity over a large range of concentrations. The final concentration of ethanol in all dilutions was less than 1%.

### 2.2. Cell Lines

Two human breast cancer cell lines (MCF-7 and HTB126), three human pancreatic cancer cells (Panc-1, Mia-Paca2, and Capan1), and a human foreskin fibroblast cell line of Hs68 were used on this study. All six cell lines were purchased from ATCC (Rockville, Md, USA). The cells were cultured and grown at 37°C and 5% CO_2_ in sterile DMEM medium with 10% fetal bovine serum (MCF-7, HTB126, Panc-1, and Hs68), DMEM with 10% fetal bovine serum and 2.5% horse serum (Mia-Paca2), and IMDM with 20% fetal bovine serum (Capan1), after the addition of glutamine and penicillin-streptomycin. Cells were grown in standard tissue culture flasks and were passaged with a solution of 0.25% trypsin-EDTA upon reaching 80% confluence. 

### 2.3. Cytotoxicity Analysis with MTT Assay

Cytotoxicity of the plant extracts on the cell lines was determined using the MTT Proliferation Assay kits from ATCC and Sigma Aldrich. The assay is based on the conversion of yellow tetrazolium salt MTT to purple formazan crystals by metabolically active cells. The amount of formazan produced is proportional to the number of viable cells. Cells were seeded in 96-well flat bottom tissue culture plates at a density of approximately 1–1.2 × 10^4^ cells/well and allowed to attach for 24 hours at 37°C. The cells were then incubated with 100 *μ*L of extracts at 100 *μ*g/mL for 24 hours. Control cultures received 100 *μ*L of medium, and blank wells without cells contained 100 *μ*L of medium. After the drug exposure period, the cells were grown for additional 24 hours in extract-free fresh medium. A volume of 10 *μ*L of the MTT reagent was then added to each well, and the plate was incubated for 4 h at 37°C. The MTT crystals were then solubilized overnight with 100 *μ*L of the MTT detergent reagent. Absorbance measurements were made at 570 nm using a Biotek HT Spectrophotometer. Cytotoxicity was expressed as the percentage of cells surviving relative to untreated cultures. All MTT experiments were performed in triplicates and repeated twice. 

### 2.4. Cytotoxicity Analysis with PC Assay

**Figure 1:**
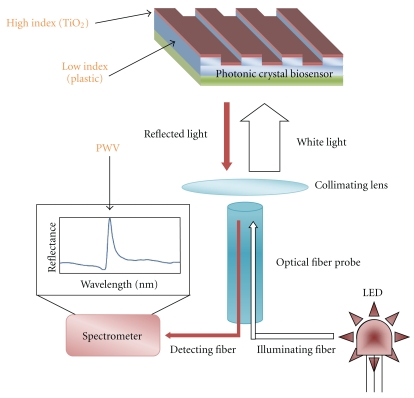
Schematic and operation of photonic crystal biosensor and readout instrument. An illuminating LED (white arrows) is normally incident to the surface of biosensor, and the reflected light (red arrows) is collected through a detecting fiber into a spectrometer, which measures the reflected peak wavelength shift from biomolecular or cellular binding.

The sensor and instrumentation of the PC assay method have been described previously in [23–27]. The biosensor measures changes in the wavelength of reflected light or peak wavelength value (PWV) as biochemical or cellular binding events take place on the surface. The imaging instrument (SRU Biosystems, Woburn, Mass, USA) illuminates the photonic crystal at normal incidence with white light, and PWV of each pixel is imaged into the entrance slit of an imaging spectrometer (Figure 1). 

**Figure 2:**
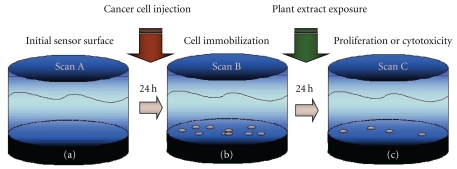
PC biosensor cancer cell imaging protocol: (a) initial baseline scan, (b) cancer cell immobilization scan after 24-hour incubation, and (c) effect of plant extracts after 24-hour incubation.

The 96-well PC biosensor microplates were utilized to confirm the cytotoxicity effects of the *Amoora* extracts on the breast cancer cell MCF-7. An initial scan with the cell culture media was taken for the background image. Approximately 500–1000 cells were seeded in the biosensor, and the cells were allowed to attach and grow for 24 hours. A second scan was performed after the 24-hour incubation. The cells were then incubated with the plant extracts at the same concentrations as the MTT assay for another 24 hours after which a final scan was performed (Figure 2). The initial cell count prior to incubation was used as a reference; thus the proliferation or cytotoxic effect can be determined. The PC cell-based assay has been published previously [11, 21, 28]. 

### 2.5. Statistical Analysis

In order to test if the cytotoxicity difference between the control and plant extracts was significant, one-way ANOVA was used to compare the different treatment groups followed by Tukeys multiple comparison tests wherever necessary. 

## 3. Results

### 3.1. In Vitro Response with the MTT Assay

**Figure 3:**
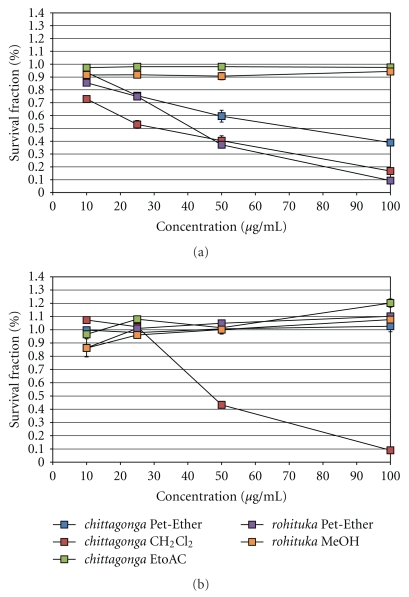
Plot of survival fraction percentage of breast cancer cells, (a) MCF-7 and (b) HTB126 cell lines, in response to the *Amoora* compounds. Cultured MCF-7 and HTB126 cells were induced with *chittagonga* (Pet-Ether, CH_2_Cl_2_, and EtoAC) and *rohituka *(Pet-Ether and MeOH) for 24 hours before performing the MTT assay. The IC_50_ values for MCF-7 in response to the *chittagonga* Pet-Ether, CH_2_Cl_2_, and *rohituka* Pet-Ether were ~42 *μ*g/mL, 48 *μ*g/mL, and 41 *μ*g/mL, respectively. For HTB126, only the *chittagonga *CH_2_Cl_2_ induced cytotoxicity at an IC_50_ of ~43 *μ*g/mL.

**Figure 4:**
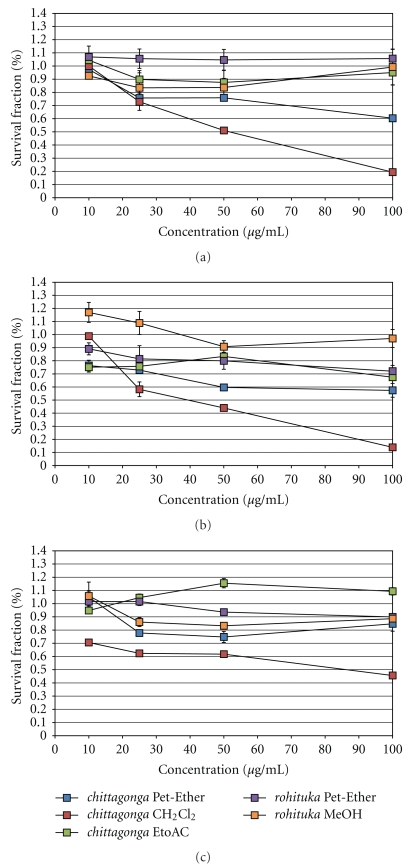
Plot of survival fraction percentage of pancreatic cancer cells, (a) Panc-1, (b) Mia-Paca2, and (c) Capan-1 cell lines, in response to the *Amoora* compounds. Cultured Panc-1, Mia-Paca2, and Capan-1 cells were induced with *chittagonga* (Pet-Ether, CH_2_Cl_2_, and EtoAC) and *rohituka *(Pet-Ether and MeOH) for 24 hours before performing the MTT assay. Out of the five *Amoora* extracts, only the *chittagonga* CH_2_Cl_2_ induced cytotoxicity in the three pancreatic cancer cell lines, with the IC_50_ values of ~39 *μ*g/mL, 30 *μ*g/mL, and 65 *μ*g/mL, respectively.

**Figure 5:**
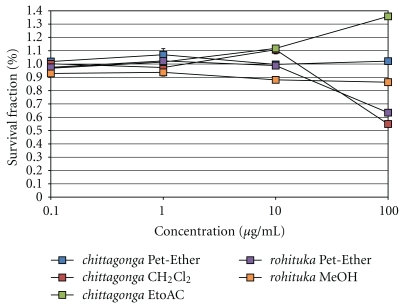
Plot of survival fraction percentage of normal fibroblast Hs68 cell line. Cultured Hs68 cells were induced with *chittagonga* (Pet-Ether, CH_2_Cl_2_, and EtoAC) and *rohituka *(Pet-Ether and MeOH) for 24 hours before performing the MTT assay. Minimal effect on the fibroblast cell line was observed from each *Amoora* species (IC_50_ > 100 *μ*g/mL).

The dose-dependent responses for the breast cancer cells are shown in Figure 3. Upon treating MCF-7 cancer cells (Figure 3(a)), the *chittagonga* species were cytotoxic and showed IC_50_ values of ~42 *μ*g/mL and 48 *μ*g/mL for the Pet-Ether and CH_2_Cl_2_ extracts, respectively. Upon treatment with the species of *rohituka*, the Pet-Ether extract showed an IC_50_ of ~41 *μ*g/mL. However, the *chittagonga* EtoAC and the *rohituka* MeOH extracts were not cytotoxic to the MCF-7 cancer cells. Treatment of HTB126 cancer cells revealed that (Figure 3(b)) only the *chittagonga* CH_2_Cl_2_ extract was cytotoxic, and here an IC_50_ value of ~43 *μ*g/mL was observed. The dose response curves for the pancreatic cancer cells are shown in Figure 4. Treatment of Panc-1, Mia-Paca2, and Capan-1 cancer cell revealed that (Figures 4(a)–4(c)) only the *chittagonga* CH_2_Cl_2_ extract showed significant cytotoxicity with IC_50_ values of ~39 *μ*g/mL, 30 *μ*g/mL, and 65 *μ*g/mL, respectively. The normal fibroblast Hs68 cells did not exhibit cytotoxic effect with any extract, indicating the potential specificity of the *Amoora* extracts against target cancer cells (Figure 5). 

For extracts that showed cytotoxic activity, the differences between the control and treatment groups were significantly different (*P* < .05). For extracts that did not suppress cell proliferation, the difference in the means is not significant compared to the control (*P* > .05), thus statistically confirming that these *Amoora* extracts are not cytotoxic at any of the above tested dose levels. 

### 3.2. In Vitro Response with the PC Assay

**Figure 6:**
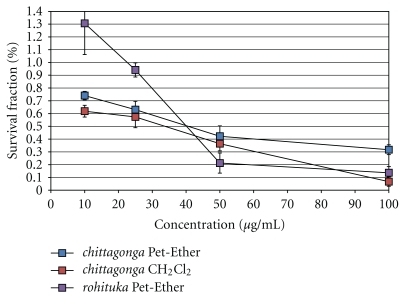
Plot of percent survival fraction of MCF-7 cells in response to *Amoora* compounds using PC biosensor assay as a verification method for the MTT assays. Cultured MCF-7 cells were induced with cytotoxic *chittagonga* (Pet-Ether and CH_2_Cl_2_) and *rohituka *(Pet-Ether) identified from the MTT assay. The IC_50_ values of the three extracts, *chittagonga* Pet-Ether, CH_2_Cl_2_, and *rohituka* Pet-Ether, are ~40 *μ*g/mL, 51 *μ*g/mL, and 38 *μ*g/mL, respectively.

For the assay employing the photonic crystal biosensor system, each well was scanned before and after treatment with the drug. Hence, for the analysis of each extract, the cell count after drug treatment was compared with the cell count of the negative control (no drug exposure). Consistent with the results obtained using the MTT assay, the Pet-Ether and CH_2_Cl_2_ fractions of *Amoora chittagonga* and Pet-Ether fraction of *Amoora rohituka* showed IC_50_ values at ~40 *μ*g/mL, 51 *μ*g/mL, and 38 *μ*g/mL, respectively (Figure 6). The other two extracts, *chittagonga* EtoAC and MeOH, did not show any cytotoxicity but instead showed proliferation of cancer cells to 4 times the control wells (data not shown).

### 3.3. Correlation of the Two Methods

**Figure 7:**
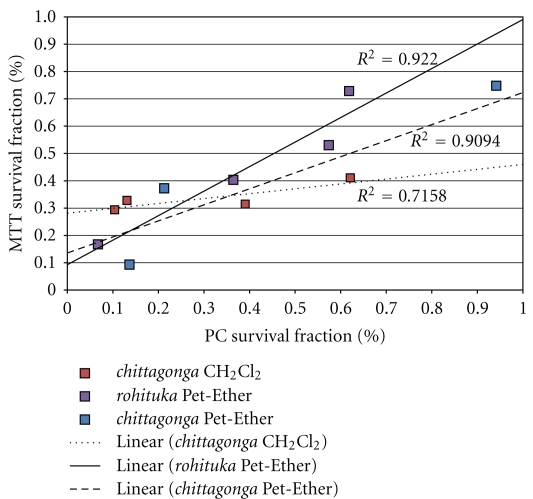
Correlation between the viability of MCF-7 cells measured using the photonic crystal assay and the MTT assay for* Amoora chittagonga* (Pet-ether and MeOH extract) and *Amoora rohituka* (Pet-ether extract), which verified cytotoxicity results obtained from the MTT assay.

The photonic crystal and the MTT assay showed good correlation for the three fractions of *Amoora* compounds that were effective against the MCF-7. The correlation plot is shown in Figure 7. For the cytotoxic fractions of *Amoora chittagonga* Pet-Ether, CH_2_Cl_2_, and *rohituka* Pet-Ether, the correlation coefficients for MCF7 cells are 0.909, 0.715, and 0.922, respectively.

## 4. Discussion

As seen from the results of the MTT and PC assays, the *Amoora* extracts induced varying levels of cytotoxicity on different cell lines within the same category. It must be noted that, for each species, all samples obtained using different extraction methods were dried and resuspended in ethanol to eliminate uncertainty that might arise from the different extraction solvents. These stock solutions were then diluted using cell culture media for the dose response studies. For the breast cancer cells, three of the extracts exhibited high cytotoxicity effect on the MCF-7 cell line, whereas only one extract was cytotoxic to the HTB126 cells. We hypothesize that the differences between MCF-7 and HTB126 may have been due to their susceptibility to *Amoora* plant extracts [29, 30]. The *chittagonga* CH_2_Cl_2 _ extract was highly cytotoxic to both breast cancer cell lines and also caused noticeable cytotoxicity to all three pancreatic cancer cell lines. All other *Amoora* extracts induced minimal levels of cytotoxicity to the pancreatic cancer cells. 

The effects of the extracts on the cancer cells are shown in Table 1. The Pet-ether of *Amoora chittagonga* affected only the MCF-7 (IC_50_ ~42 *μ*g/mL) and had little or no effect on the other cancer cell lines. The *Amoora chittagonga* CH_2_Cl_2_ affected MCF-7 (~48 *μ*g/mL), HTB126 (~43 *μ*g/mL), Panc-1 (~39 *μ*g/mL), Mia-Paca2 (30 *μ*g/mL), and Capan-1 (~65 *μ*g/mL) cell lines. Although the IC_50_ values differ between the effective extracts of *Amoora chittagonga*, we calculated the significance of the cytotoxicity for MCF-7 and showed that the two fractions did not significantly differ in their cytotoxic potential (*P* > .05). The Ethyl acetate (EtoAC) partitionate of *Amoora chittagonga* did not affect the proliferation of any of the cancer cell lines as seen from the MTT results. The efficacy of the ethyl acetate partitionate was significantly different from that of the Petroleum ether and the CH_2_Cl_2_ fractions (*P* < .05). The *rohituka* Pet-Ether extract was effective against MCF-7 (consistent with the previous literature [31]), but not the other cell lines. Two independent fractions of *Amoora rohituka* differed significantly in their effectiveness (*P* < .05), wherein the petroleum ether extract was highly cytotoxic against the cancer cells and the MeOH extract had no effect on the cell proliferation. The varying results from a single species of *Amoora* plant suggest that the partitioning of plant extracts using different solvents may have a significant influence on the biological activity of the resulting fraction. Only *chittagonga* CH_2_Cl_2_ induced cytotoxicity in all five cancer cell lines. We hypothesized that the difference between the cytotoxicity of *rohituka *and *chittagonga *may have been caused by the content of amooranin within the extracts. Most of the current studies involving *Amoora rohituka *extract showed considerable antitumor effect, but it seemed that *Amoora chittagonga *would also be an attractive extract to further explore in depth its anticancer properties. The cytotoxicity difference between the five cancer cell lines and normal cell line suggested that *Amoora* plants extracts may affect specific cancer cell lines. 

In order to rapidly examine a group of cancer cell lines against a library of plant extracts, it is necessary to utilize a high-throughput screening technology. The testing of the extracts could be easily facilitated using the photonic crystal sensor assay, which senses attachment of viable cells and produces a signal that can be quantified in terms of the cell numbers. If the cells become apoptotic or necrotic in response to the concentration of the drug, they lose attachment with the sensor surface, and this results in a weak or no signal at the point of attachment. The advantage conferred by this method is that a vast library of compounds can be simultaneously tested without the need for any fluorescent labeling markers or other colorimetric assays. The PC biosensor assay is used to verify the cytotoxicity effect of *Amoora *extracts identified by the MTT assay. The results of the PC assay correlate closely with that of the MTT assay performed under similar experimental conditions. In addition, the PC biosensor assay is able to observe label-free images of proliferation and cytotoxicity of clusters of cancer cells in a well from the *Amoora* plant extracts, whereas the MTT assay only provides a bulk colorimetric response for a given well. The PC assay was performed on only MCF-7 cell line to show the capability in detecting cytotoxic effect of plant extracts compared to MTT assay. We have previously demonstrated the use of the PC method for accurate high-throughput screening of a library of plant extracts [28]. Based on our results, a study of the active ingredients in the *Amoora* chittagonga plant extract that are responsible for cancer-specific cytotoxicity effects is warranted. 

## 5. Conclusion

In this work, we have further explored the therapeutical effect of the *Amoora* plant extracts against five cancer cell lines. Although numerous studies have reported medicinal and therapeutic effects of the *Amoora* extracts, it is important to examine the effects of unreported *Amoora* species against specific cancer cell lines in an effort to identify preliminary candidates for alternative cancer therapeutics. By employing the MTT assay, various cytotoxicity effects were observed for a combination of five *Amoora* extracts and five cancer cell lines. Interestingly, when samples of a species of the *Amoora* plant were extracted using different extraction methods, we found that these crude extracts showed varying levels of cytotoxicity on the cancer cells. By identifying the active *Amoora* extracts on specific cancer cell lines, further research could be performed on examining the biochemical compositions to fully understand the mechanism of the cytotoxicity effects. 

##  Conflict of Interests

B. T. Cunningham declares a duality of interest. The photonic crystal biosensors used in the paper are made by a company, SRU Biosystems, that B. T. Cunningham cofounded in June, 2000. B. T. Cunningham is currently the Chief Technical Officer of SRU Biosystems and own shares of the company stock. His full-time employment is with the University of Illinois in Urbana-Champaign, where he is an Associate Professor. 

## Figures and Tables

**Table 1:** IC_50_ values and effects of five *Amoora* plant extracts on the six cell lines. Note that only normal fibroblast cell line has no effect for each extract.

	MCF-7	HTB126	Panc-1	Mia-Paca2	Capan-1	Hs68
*chittagonga* Pet-Ether	42 *μ*g/mL	No Effect	No Effect	No Effect	No Effect	No Effect
*chittagonga* CH_2_Cl_2_	48 *μ*g/mL	43 *μ*g/mL	39 *μ*g/mL	30 *μ*g/mL	65 *μ*g/mL	No Effect
*chittagonga* EtoAC	No Effect	No Effect	No Effect	No Effect	No Effect	No Effect
*rohituka* Pet-Ether	41 *μ*g/mL	No Effect	No Effect	No Effect	No Effect	No Effect
*rohituka* MeOH	No Effect	No Effect	No Effect	No Effect	No Effect	No Effect

## References

[1] Edwards BK, Ward E, Kohler BA (2010). Annual report to the nation on the status of cancer, 1975–2006, featuring colorectal cancer trends and impact of interventions (risk factors, screening, and treatment) to reduce future rates. *Cancer*.

[2] (2009). Cancer Facts and Figures 2009. *American Cancer Society*.

[3] (2007). What you need to know about cancer of the pancreas. *National Cancer Institute*.

[4] (2009). Pancreatic cancer. *National Cancer Institute, U.S. National Institutes of Health*.

[5] Goldstein MS (2003). Complementary and alternative medicine: its emerging role in oncology. *Journal of Psychosocial Oncology*.

[6] Rabi T, Gupta RC (1995). Antitumor and cytotoxic investigation of *Amoora rohituka*. *International Journal of Pharmacognosy*.

[7] Rabi T (1996). Antitumour activity of amooranin from *Amoora rohituka* stem bark. *Current Science*.

[8] Kirtikar KR, Basu BD (1980). *Indian Medicinal Plants*.

[9] Ghani A (1998). *Medicinal Plants of Bangladesh: Chemical Constituents and Uses*.

[10] Chowdhury R, Rashid RB (2003). Effect of the crude extracts of *Amoora rohituka* stem bark on gastrointestinal transit in mice. *Indian Journal of Pharmacology*.

[11] Chan LL, Gosangari SL, Watkin KL, Cunningham BT (2007). A label-free photonic crystal biosensor imaging method for detection of cancer cell cytotoxicity and proliferation. *Apoptosis*.

[12] Rabi T, Karunagaran D, Krishnan Nair M, Bhattathiri VN (2002). Cytotoxic activity of amooranin and its derivatives. *Phytotherapy Research*.

[13] Rabi T, Ramachandran C, Fonseca HB (2003). Novel drug amooranin induces apoptosis through caspase activity in human breast carcinoma cell lines. *Breast Cancer Research and Treatment*.

[14] Ramachandran C, Nair PKR, Alamo A, Cochrane CB, Escalon E, Melnick SJ (2006). Anticancer effects of amooranin in human colon carcinoma cell line in vitro and in nude mice xenografts. *International Journal of Cancer*.

[15] Ramachandran C, Rabi T, Fonseca HB, Melnick SJ, Escalon EA (2003). Novel plant triterpenoid drug amooranin overcomes multidrug resistance in human leukemia and colon carcinoma cell lines. *International Journal of Cancer*.

[16] Polonsky J, Varon Z, Marazano C (1979). The structure of amoorastatone and the cytotoxic limonoid 12-hydroxyamoorastatin. *Experientia*.

[17] Ren W, Qiao Z, Wang H, Zhu L, Zhang L (2003). Flavonoids: promising anticancer agents. *Medicinal Research Reviews*.

[18] Chumkaew P, Kato S, Chantrapromma K (2006). Potent cytotoxic rocaglamide derivatives from the fruits of Amoora cucullata. *Chemical and Pharmaceutical Bulletin*.

[19] Rahman MS, Rahman MZ, Wahab MA, Chowdhury R, Rashid MA (2008). Antimicrobial activity of some indigenous plants of Bangladesh. *Dhaka University Journal of Pharmaceutical Sciences*.

[20] Maxwell JF (1986). Vascular flora of Khao Khieo wildlife sanctuary chonburi province Thailand. *Natural History Bulletin of Siam Society*.

[21] Lin B, Li P, Cunningham BT (2006). A label-free biosensor-based cell attachment assay for characterization of cell surface molecules. *Sensors and Actuators. B*.

[22] Chowdhury R, Hasan CM, Rashid MA (2003). Antimicrobial activity of Toona ciliata and *Amoora rohituka*. *Fitoterapia*.

[23] Cunningham B, Li P, Lin B, Pepper J (2002). Colorimetric resonant reflection as a direct biochemical assay technique. *Sensors and Actuators. B*.

[24] Haes AJ, Van Duyne RP (2002). A nanoscale optical biosensor: sensitivity and selectivity of an approach based on the localized surface plasmon resonance spectroscopy of triangular silver nanoparticles. *Journal of the American Chemical Society*.

[25] Joannopoulos JD, Meade RD, Winn JN (1995). *Photonic Crystals*.

[26] Munk BA (2000). *Frequency Selective Surfaces*.

[27] Cunningham BT, Li P, Schulz S (2004). Label-free assays on the BIND system. *Journal of Biomolecular Screening*.

[28] Chan LL, Gosangari SL, Watkin KL, Cunningham BT (2008). Label-free imaging of cancer cells using photonic crystal biosensors and application to cytotoxicity screening of a natural compound library. *Sensors and Actuators. B*.

[29] Ford J, Jiang M, Milner J (2005). Cancer-specific functions of SIRT1 enable human epithelial cancer cell growth and survival. *Cancer Research*.

[30] Rivenbark AG, Jones WD, Coleman WB (2006). DNA methylation-dependent silencing of CST6 in human breast cancer cell lines. *Laboratory Investigation*.

[31] Rabi T, Gupta RC, Gulati AK (1994). Influence of *Amoora rohituka* on MCF-7 human mammary adenocarcinoma cells in vitro. *Indian Journal of Pharmaceutical Sciences*.

